# The Challenges of Treating Patients with Breast Cancer and Obesity

**DOI:** 10.3390/cancers15092526

**Published:** 2023-04-28

**Authors:** Alexis LeVee, Joanne Mortimer

**Affiliations:** Department of Medical Oncology, City of Hope Comprehensive Cancer Center, Duarte, CA 91010, USA; jmortimer@coh.org

**Keywords:** obesity, breast cancer, systemic treatment, outcomes

## Abstract

**Simple Summary:**

Obesity is a poor prognostic factor for patients with breast cancer, resulting in increased risk of recurrence and death due to breast cancer. Obesity can affect both the efficacy and toxicity of systemic cancer therapies, including chemotherapy, endocrine therapy, immunotherapy, and targeted therapies. In this review, we summarize the impact of obesity on the clinical outcomes of systemic therapies in patients with breast cancer, describe the molecular mechanisms through which obesity can affect systemic therapies, and highlight additional considerations for treating patients with obesity and breast cancer. Further research that focuses on the clinical outcomes in patients with obesity is needed to guide treatment decision-making.

**Abstract:**

Obesity is defined as a body mass index (BMI) of 30 kg/m^2^ or more and is associated with worse outcomes in patients with breast cancer, resulting in an increased incidence of breast cancer, recurrence, and death. The incidence of obesity is increasing, with almost half of all individuals in the United States classified as obese. Patients with obesity present with unique pharmacokinetics and physiology and are at increased risk of developing diabetes mellitus and cardiovascular disease, which leads to specific challenges when treating these patients. The aim of this review is to summarize the impact of obesity on the efficacy and toxicity of systemic therapies used for breast cancer patients, describe the molecular mechanisms through which obesity can affect systemic therapies, outline the existing American Society of Clinical Oncology (ASCO) guidelines for treating patients with cancer and obesity, and highlight additional clinical considerations for treating patients with obesity and breast cancer. We conclude that further research on the biological mechanisms underlying the obesity–breast cancer link may offer new treatment strategies, and clinicals trials that focus on the treatment and outcomes of patients with obesity and all stages of breast cancer are needed to inform future treatment guidelines.

## 1. Introduction

Obesity, defined as a body mass index (BMI) of 30 kg/m^2^ or more, is one of the world’s largest health problems. The World Health Organization has declared obesity as a global epidemic with more than one billion people worldwide classified as obese [1]. According to the National Health and Nutrition Examination Survey, 41.9% of the population in the United States is obese [2]. Along with the rising rates of obesity, the incidence of breast cancer is also increasing by approximately 0.5% per year [3]. Obesity and breast cancer are strongly linked, with numerous studies demonstrating that obesity adversely impacts both breast cancer incidence and outcomes [4,5,6]. Postmenopausal women with obesity have a 52% increased risk of hormone receptor (HR)-positive breast cancer compared to women with normal weight, and the risk increases to 86% for women with a BMI greater than 35 kg/m^2^ [4] Postmenopausal women with obesity are also more likely to present with more aggressive and advanced disease at diagnosis, including larger tumors, positive lymph nodes, and regional or distant stage [4]. On the other hand, obesity does not increase the risk of estrogen receptor-negative or progesterone receptor-negative breast cancer in postmenopausal women [4], and reduces the risk of breast cancer in premenopausal women [7], which may be the result of differences in estrogen and adipokine-driven signaling pathways. However, after breast cancer diagnosis, obesity results in worse overall survival (OS) in all breast cancer subtypes (HR+ HER2- subtype hazard ratio (HR) = 1.39, 95% confidence interval (CI) 1.20–1.62, *p* < 0.001; HER2+ subtype HR = 1.18, 95% CI 1.05–1.33, *p* = 0.006; and triple-negative subtype HR = 1.32, 95% CI 1.13–1.53, *p* < 0.001) [8], and worse OS in both premenopausal (relative risk (RR) = 1.75, 95% CI 1.26–2.41) and postmenopausal women (RR = 1.34, 95% CI 1.18–1.53) [9].

Obesity contributes to the development of cardiovascular disease risk factors, including dyslipidemia, type 2 diabetes, and hypertension, leading to an increased risk of cardiovascular mortality [10]. Women with breast cancer are at an even higher risk of developing and dying of cardiovascular disease compared to women without breast cancer due to a number of mechanisms including cancer-related treatment exposure and shared risk factors for cancer and cardiovascular disease [11]. Ultimately, women with obesity and breast cancer have a 60% increased risk of dying of cardiovascular disease after breast cancer diagnosis compared to women with normal weight, and a 41% increased risk of dying of all-cause mortality [9]. Given that patients with obesity suffer from worse clinical outcomes, these patients present with unique challenges during breast cancer treatment.

The pathophysiology underlying the obesity–breast cancer link is complex and multifactorial, involving mechanisms associated with increased circulating insulin and glucose, altered levels of adipokines and estrogen signaling, and chronic inflammation [12,13]. Obesity is associated with metabolic dysfunction and hyperinsulinemia, resulting in increased synthesis of insulin-like growth factor-1 (IGF-1), which activates the PI3K and MAPK signaling pathways, leading to cancer cell proliferation and survival. Obesity also leads to dysfunctional adipocytes, the major cellular component in adipose tissue, which can release adipokines, cytokines, and metabolic substrates to further promote breast cancer oncogenesis and progression [14]. High levels of circulating estrogen are also observed in patients with obesity and are associated with an increased risk of HR-positive breast cancer due to estrogen-mediated alterations in cellular metabolism and signaling pathways [12]. Additionally, obesity leads to a state of chronic, low-grade inflammation, which further contributes to insulin resistance and promotes tumorigenesis. Furthermore, obesity can alter the microbiome, which may impact breast cancer risk and the efficacy of breast cancer therapies [12,15,16,17]. Treatment-related issues in patients with obesity may also be one of the key factors underlying the inferior outcomes in patients with obesity and breast cancer.

A better understanding of how these molecular mechanisms impact the biology of breast cancer in patients with obesity is needed to inform future therapeutic trials. Given the increasing prevalence of obesity and the urgent need to treat these patients appropriately, the American Society of Clinical Oncology (ASCO) published clinical practice guidelines on the appropriate chemotherapy dosing strategy for patients with cancer and obesity in 2012, and more recently published updated guidelines in 2021 to include immunotherapy and targeted cancer therapy dosing in this patient population [18,19]. In this review, we summarize available data that address the unique issues related to the delivery of systemic therapy in patients with obesity and breast cancer, review the ASCO practice guidelines on treatment strategies in patients with obesity, and provide clinical considerations specific to breast cancer treatment with the goal of improving outcomes in this high-risk population.

## 2. Systemic Chemotherapy

### 2.1. Chemotherapy Dosing and Toxicity

In the adjuvant setting, chemotherapy dose intensity is critical to maximize the chance of long-term survival [20]. The dose of most chemotherapy agents is determined by a patients’ estimated body surface area (BSA), which incorporates measures of body weight and height into several validated formulas, and is based on the assumption that pharmacological processes are related to body size. However, BSA-based dosing formulas were not developed for use in patients who are obese or morbidly obese. As a result, BSA-based formulas provide inconsistent measures of BSA when used in patients with obesity compared to patients of average-build [21]. BSA-based formulations also do not take into account body composition, which is characterized by the distribution of lean and fat tissues throughout the body, or individual metabolism and excretion factors, which can further limit the estimates of BSA-based formulas in providing a true assessment of drug pharmacokinetics in patients with obesity.

BSA-based dosing of chemotherapy is currently the standard for most chemotherapy, despite significant limitations. Although select cytotoxic agents are prescribed at a fixed-dose independent of weight or BSA, there is limited evidence that fixed-dosing strategies for other chemotherapeutic agents are equivalent to BSA-based dosing. For example, a fixed dose of capecitabine has been developed for use in breast cancer, but a study that evaluated fixed-dose capecitabine compared to BSA-based dosing for patients with breast cancer, colorectal cancer, gastric cancer, and other cancers showed similar rates of toxicity and efficacy of fixed-dose capecitabine compared to BSA-based dosing, including for patients categorized in a high BSA group [22]. Other dosing strategies of chemotherapy are also being explored in breast cancer. For example, the phase III Pan-European Tailored Chemotherapy (PANTHER) study investigated a dose-dense (DD) chemotherapy and tailoring strategy according to hematologic toxicity of epirubicin/cyclophosphamide (E/C) and tailored docetaxel (D) to standard interval 5-fluorouracil/E/C and D for patients with high-risk early breast cancer [23]. In a secondary analysis of the study, patients with obesity treated with the dose tailoring strategy were found to have improved relapse-free survival (HR = 0.51, 95% CI 0.30–0.89, *p* = 0.02) without increased toxicities compared to patients without obesity [24]. Additional studies that address individually tailored chemotherapy dosing are needed.

Although current guidelines recommend BSA-based dosing, studies show that almost 40% of patients with obesity receive reduced doses of chemotherapy [25]. Physicians may underdose patients with obesity due to concerns of dose-dependent toxicity and fears that BSA-based formulas may overestimate drug distribution in these patients. Frequently in clinical practice, chemotherapy doses are capped at a BSA of 2.0 m^2^ or adjusted to an ideal body weight for patients with an elevated BMI due to safety concerns. When dose adjustments are made, patients with obesity ultimately receive lower doses than is recommended, which translates to worse breast cancer outcomes. A secondary analysis of the CALGB adjuvant trial 8541 found that patients with obesity who started cycle 1 of chemotherapy with drugs reduced to <95% of the weight-based dose had shorter failure-free survival rates (overall adjusted failure risk ratio = 0.73, 95% CI 0.53–1.00) compared to patients with obesity who received chemotherapy dosing within 5% of the expected dose based on actual weight [26]. The CALGB 8541 trial also found that any patient who received a reduced total dose and dose intensity of chemotherapy experienced higher rates of recurrence and increased mortality [27]. The theoretical concern that increased adjuvant chemotherapy dosing will lead to increased toxicity has been dispelled in multiple systemic reviews of the literature evaluating toxicity of chemotherapy in patients with obesity. Full weight-based dosing of chemotherapy in patients with obesity is associated with similar or less toxicity [28,29]. 

### 2.2. The Impact of Obesity on Chemotherapy Efficacy

Even with appropriate dosing of adjuvant chemotherapy, patients with obesity have worse breast cancer outcomes. The combined adjuvant clinical trials (E1199, E5188, and E3189) enrolled over 6000 women and found that women with obesity and HR-positive, HER2-negative breast cancer have worse disease-free survival (DFS) (HR = 1.24, 95% CI 1.06–1.46, *p* = 0.0008) and OS outcomes (HR = 1.37, 95% CI 1.13–1.67, *p* = 0.002) compared to women with normal weight [30]. Given the trials used appropriate BSA-based dosing and included only patients with normal organ function, this suggests that the inferior outcomes were likely attributable to obesity alone. A retrospective analysis of the adjuvant BIG 2-98 trial (n = 2887) comparing non-docetaxel to docetaxel-containing chemotherapy showed that patients with obesity had reduced DFS (HR = 1.32, 95% CI 1.08–1.62, *p* = 0.007) and OS (HR = 1.63, 95% CI 1.27–2.09, *p* < 0.001) in the docetaxel group; however, no difference in DFS (HR 1.11, 95% CI 0.83–1.47, *p* = 0.49) or OS (HR 1.10, 95% CI 0.78–1.54, *p* = 0.59) was observed in the non-docetaxel group [31]. To determine whether this finding could be attributed to patients with obesity receiving a reduced dosing regimen, a separate analysis showed that the results remained when considering only patients who received a relative dose intensity ≥ 85% for docetaxel. The authors suggest that these findings may be due to the lipophilic nature of docetaxel that results in a higher volume of distribution, which may translate to decreased efficacy in patients with a higher BMI. Patients who are overweight and obese with HR-positive/HER2-positive disease treated in the NeoALTTO trial also had decreased rates of pCR (odds ratio (OR) = 0.55, 95% CI 0.30–1.01, *p* = 0.053), while no difference in pCR (OR = 1.30, 95% CI 0.76–2.23, *p* = 0.331) was observed in HR-negative patients [32]. The authors suggest that the lower pCR rate observed in HER2-positive, HR-positive breast cancer patients may be due to the association with obesity and increased estrogen signaling pathways in HR-positive breast cancer, resulting in increased tumor proliferation and a worse response to neoadjuvant therapy. Additional investigation is needed into whether the HR status is a predictor for chemotherapy outcomes in patients with obesity. 

### 2.3. Weight Gain following Chemotherapy

Chemotherapy can directly or indirectly lead to weight gain through the induction of menopause, which may further exacerbate the poor health outcomes in patients with obesity. Although weight gain was more common with outdated chemotherapy regimens (e.g., cyclophosphamide, methotrexate, and 5-fluorouracil [CMF]), a recent meta-analysis of 25 papers showed that the average weight change in studies published after 2000 is 1.3 kg [33]. Pre-menopausal patients appear to be at increased risk of weight gain after adjuvant therapy [34]. In an observational study of 86 women treated with a 12- to 18-week course of (neo)adjuvant chemotherapy, chemotherapy was also shown to negatively impact lipid profiles and promote the development of insulin resistance in addition to an increase in weight [35]. 

Although BMI at diagnosis is a predictor for worse breast cancer outcomes, it is not known if weight gain after breast cancer diagnosis leads to worse breast cancer outcomes [36]. In a meta-analysis, which included 7 prospective cohorts and 2 clinical trials of over 23,000 breast cancer survivors, weight gain of 10% or more after breast cancer diagnosis was associated with increased breast cancer-specific mortality and all-cause mortality, but was not associated with increased risk of recurrence [36]. In a pooled analysis of 4 prospective cohorts of over 18,000 breast cancer survivors, post-diagnosis weight gain was associated with increased risk of late recurrence, but there was no association with all-cause mortality [37]. Nonetheless, post-diagnosis weight gain may impact the success of subsequent cancer treatments. For example, increased BMI is associated with increased toxicity during radiation treatment and increased complications during reconstructive surgery [38,39]. Post-diagnosis weight can also contribute to poor quality of life and low self-esteem and increases the risk of development of comorbid conditions, which can each negatively impact the overall health in breast cancer patients [40]. 

## 3. Endocrine Therapy

### 3.1. The Impact of Obesity on Choice and Duration of Adjuvant Endocrine Therapy

Endocrine therapy dosing is fixed regardless of individual patient factors; however, current evidence suggests that there may be differences in outcomes with the use and type of endocrine therapy in patients with obesity. Because endocrine therapy targets estrogen and as obesity is associated with increased estrogen production, there is concern that estrogen therapy may have reduced effectiveness in patients with obesity. Patients with obesity have increased estrogen levels as a result of increased breast adipose tissue, which is a major site of aromatase activity [41]. The main sources of estrogen, estrone and estradiol, are converted from androgens in breast adipose tissue; therefore, an increase in aromatase activity in the breast tissue results in increased estrogen [41]. Obesity also leads to a decrease in sex hormone-binding globulin that inhibits estradiol, resulting in an additional increase in estrogen [42].

Aromatase inhibitors (AIs) block the aromatization of estrogens from androgens, while tamoxifen binds to estrogen receptors producing both estrogenic and anti-estrogen effects. Given that patients with obesity have increased levels of estrogen, there is a concern that endocrine therapy may not adequately suppress serum estrogen levels in breast cancer survivors with obesity, which may result in an increased risk of relapse. Several studies examined levels of serum estrogen before and after treatment with AIs. These studies confirm that patients with obesity at baseline have higher estradiol levels—with patients with BMI > 35 kg/m^2^ having almost three times higher estradiol levels compared to patients with BMI < 25 kg/m^2^. The results show that serum levels of estradiol are higher after treatment with AIs in patients with obesity compared to patients without obesity [43,44]. 

Obesity also leads to a number of other endocrine resistance mechanisms. Obesity leads to dysregulated adipocytes, which secrete an imbalance of adipokines (such as leptin and insulin), metabolites (such as cholesterol and free fatty acids), and cytokines (such as TNFα and interleukins), which can induce endocrine therapy resistance by activating various signal transduction pathways and regulating apoptosis-related genes [14,45]. Certain adipokines and cytokines can also modulate estrogen synthesis by upregulating aromatase gene activity and have been found to diminish the efficacy of endocrine therapy in vitro [14,45]. Patients with obesity also have higher insulin levels, leading to increased IGF-1 in breast cancer cells, which activates the PI3K/AKT/mTOR and RAS/RAF/MAPK signaling pathways that result in endocrine therapy resistance [13,46,47]. Furthermore, obesity is associated with the overproduction of proinflammatory molecules and reactive oxygen species, which can further promote tumor progression despite hormone therapy [45,48,49,50].

Several large studies have addressed whether obesity predicts inferior clinical outcomes in patients treated with endocrine therapy. In a study of over 18,000 women treated for early-stage breast cancer within the Danish Breast Cancer Cooperative Group with 30 years follow-up, patients with obesity treated with adjuvant endocrine therapy with either tamoxifen or AIs had worse breast cancer outcomes with an increased risk of distant metastases (HR = 1.46, 95% CI 1.11–1.92, *p* = 0.007) and breast cancer-related death (HR = 1.38, 95% CI 1.11–1.71, *p* = 0.003) compared to patients with normal weight [5]. However, four large randomized clinical trials that compared an AI to tamoxifen in the adjuvant setting showed mixed results regarding the effect of BMI on treatment efficacy. In the ATAC trial, which investigated anastrozole, tamoxifen, or the combination in postmenopausal women with early-stage breast cancer, women with a high BMI (BMI > 35 kg/m^2^) had more recurrences than women with a normal BMI (HR = 1.39; 95% CI 1.06–1.82, *p* = 0.03) after 100 months of follow-up [51]. Breast cancer survivors who received anastrozole had a 27% decreased recurrence rate than those on tamoxifen; however, the benefit of anastrozole was greater in women with a lower BMI, whereas tamoxifen was effective across all BMIs [51]. Similarly, in a secondary analysis of the prospective ABCSG-12 trial, which investigated the efficacy of ovarian suppression with goserelin in combination with anastrozole or tamoxifen with or without zoledronic acid in premenopausal women, overweight patients on anastrozole had a 60% increased risk of disease recurrence (HR = 1.60, 95% CI 1.06–2.41, *p* = 0.02) and more than a doubling in the risk of death (HR = 2.14, 95% CI 1.17–3.92, *p* = 0.01) compared to patients with normal weight treated with anastrozole [52]. Overweight patients treated with anastrozole had almost a 1.5-fold increased risk of disease recurrence (HR = 1.49, 95% CI 0.93–2.38, *p* = 0.08) and a 3-fold increased risk of death (HR = 3.03, 95% CI 1.35–6.82, *p* = 0.004) compared with overweight patients treated with tamoxifen [52]. In the BIG 1-98 trial that compared tamoxifen versus letrozole in postmenopausal women, patients with obesity had slightly worse OS (HR = 1.19, 95% CI 0.99–1.44) than patients with normal BMI, but there was no difference in the treatment effect according to BMI (*p* = 0.74) [53]. Lastly, in the TEAM trial that compared tamoxifen followed by exemestane versus exemestane monotherapy for 5 years in postmenopausal women, exemestane for 2.75 years was associated with a reduced risk of relapse in patients with obesity (HR 0.57, 95% CI 0.39–0.84, *p* = 0.004) compared to tamoxifen for 2.75 years, but no difference in disease recurrence was seen between the two groups at 5 years (HR = 0.75, 95% CI 0.56–1.01, *p* = 0.058) [54].

Given that the ATAC and ABCSG-12 trials both showed decreased efficacy of anastrozole in patients with obesity, the question arises whether an increased dose of AIs may be more effective for these patients. Although early phase III clinical trials comparing 1 mg anastrozole to 10 mg anastrozole found no difference in efficacy, these studies were not powered to determine differences based on BMI [55]. Additional research is needed to test whether higher doses of AIs will lead to increased estrogen suppression and improved outcomes. The clinical trial “Impact of Obesity on the Efficacy of Endocrine Therapy with Aromatase Inhibitors” (NCT01758146) is currently evaluating the efficacy of adjuvant tamoxifen compared with letrozole and should provide more information on endocrine therapy selection in this important population.

To our knowledge, no study has evaluated whether obesity impacts the benefit from extended endocrine therapy. In the MA.17R and ATLAS trials, respectively, longer duration of AIs and tamoxifen were shown to reduce the risk of recurrence and breast cancer mortality [56,57]. Women with high-risk tumors may benefit from extended endocrine therapy, but this benefit needs to be weighed against the side effects and risks of treatment. For a breast cancer survivor with obesity, consideration of the comorbidities and cardiac risk factors is warranted prior to the choice and duration of endocrine therapy. 

### 3.2. The Impact of Obesity on Endocrine Therapy in Metastatic Disease

The impact of BMI on the efficacy of endocrine therapy in the metastatic setting has been less well-studied. Two retrospective studies that investigated AIs and fulvestrant for metastatic HR-positive breast cancer showed no difference in efficacy according to BMI [58,59]. Conversely, in another retrospective study of 105 women with advanced breast cancer treated with fulvestrant during any line of therapy, patients who are overweight and obese had almost a 2.5-fold decreased clinical benefit rate (defined as the proportion of partial or complete responses or stable disease lasting at least 6 months) when treated with fulvestrant compared to patients with normal weight (*p* < 0.001) irrespective of the estrogen receptor expression [60]. Similarly, in an observational study of a historic cohort of HR-positive metastatic breast cancer patients treated with fulvestrant, a higher BMI was associated with a shorter PFS (HR = 1.89, 95% CI 1.11–3.24, *p* = 0.02) in patients who showed prior hormone resistance [61]. Because single-agent endocrine therapy is no longer first-line therapy, it is hard to put the aforementioned data into context. However, given the reduced efficacy noted in patients with obesity in the adjuvant trials, it is reasonable to test whether the same is true in metastatic disease.

### 3.3. Toxicities of Endocrine Therapy

Data investigating the impact of BMI on toxicity with endocrine therapy are limited [62]. One retrospective study of 484 patients with early-stage breast cancer treated with tamoxifen, AIs, and ovarian ablation showed that obesity was associated with more than a two-fold increased odds of experiencing endocrine-related side effects (OR 2.29, 95% CI 1.04–5.07, *p* = 0.04), which led to increased rates of early treatment change or discontinuation compared to patients with normal weight [63]. In another retrospective, exploratory analysis of the ATAC trial that investigated adjuvant therapy with anastrozole, tamoxifen, or the combination, women with obesity were found to have more joint symptoms than women with normal weight (OR = 1.32, 95% CI 1.14–1.53) for both types of endocrine therapy [64]. 

Although AIs and tamoxifen are generally well-tolerated, endocrine therapy can lead to a host of cardiometabolic adverse effects. In one meta-analysis, AIs were associated with a slightly increased risk of cardiovascular events compared to tamoxifen; however, the results were not statistically significant (OR = 1.21, 95% CI 0.99–1.48). In addition, AIs tended to have a higher risk for dyslipidemia (OR = 2.24, 95% CI 0.99–5.06) [65]. In comparison, AIs are associated with a 41% decreased risk of venous thromboembolism compared to tamoxifen, and the risk of venous thromboembolism with tamoxifen is increased for patients with a BMI ≥ 25 [66,67]. Given patients with obesity are at increased risk of both endocrine therapy-related side effects and cardiovascular events, patients with obesity on endocrine therapy should be carefully monitored for toxicities and compliance with adjuvant therapy. 

## 4. HER2-Targeted Treatments

### 4.1. Trastuzumab Dosing and Efficacy

Trastuzumab, a monoclonal antibody that targets the HER2 transmembrane growth factor receptor, is a standard treatment for HER2-positive breast cancer. Trastuzumab is dosed according to body weight when an intravenous route is used based on phase I dose escalation studies that utilized a weight-based dosing schedule [68,69]. In a secondary analysis of the N9831 trial that compared chemotherapy with or without trastuzumab in over 3400 women with HER2-positive, early-stage breast cancer, women with obesity had a decreased DFS (HR 1.31, 95% CI 1.07–1.59) compared to women with normal weight [70]. However, after stratification according to the type of treatment received, there was no difference in the DFS of women who are overweight and those who are obese compared to women with normal weight after treatment with IV adjuvant trastuzumab. This study suggests that IV adjuvant trastuzumab may improve clinical outcomes regardless of BMI, although the study was insufficiently powered to detect a statistical significance according to the treatment received [70]. 

A fixed-dose subcutaneous (SC) formulation of trastuzumab is available at 600 mg per dose based on subsequent pharmacokinetic studies that reported a minimal effect of body weight on trastuzumab exposure [71]. The HannaH study compared (neo)adjuvant trastuzumab once every 3 weeks with either 600 mg SC or 8 mg/kg IV loading dose followed by 6 mg/kg maintenance for early HER2-positive breast cancer and showed non-inferiority of SC versus IV trastuzumab [72]. However, further population pharmacokinetic models show that the first cycle of treatment with 600 mg SC trastuzumab may not reach the target plasma concentration in patients with an elevated BMI [73]. This suggests that a weight-adjusted IV dosage may be required in the first cycle, or that an additional loading dose is required when switching from IV to SC dosing [73,74]. Nevertheless, current US prescribing guidelines do not require modifications according to weight given that SC trastuzumab achieved equal or higher concentration immediately before the next dose is administered (C_trough_) at pre-dose cycle 8 with no difference in efficacy across body groups compared to IV trastuzumab [75,76].

### 4.2. Trastuzumab Toxicity

Trastuzumab can cause cardiotoxicity in a small percent of patients, characterized by asymptomatic decreases in the left ventricular ejection fraction (LVEF) and less often congestive heart failure [77]. Cardiotoxicity can occur independent of the cumulative doses of trastuzumab, and LVEF recovery to baseline often occurs following trastuzumab discontinuation. In a meta-analysis of 8745 women with breast cancer treated with anthracyclines and sequential anthracyclines and trastuzumab, Guenancia et al. reported that women who are overweight and those who are obese are at increased risk of developing cardiotoxicity from trastuzumab plus chemotherapy (OR = 1.38, 95% CI 1.06–1.80) compared to women with normal weight [78]. In an exploratory analysis of the ALTTO BIG 2-06 trial, which investigated trastuzumab and/or lapatinib as adjuvant treatment for early stage HER2-positive disease, patients with obesity had a higher incidence of grade 3/4 adverse events (*p* < 0.001) and serious adverse events (*p* < 0.001), which led to a statistically significant increase in treatment discontinuation (*p* < 0.001) [79]. These data suggest that patients with obesity and HER2-positive breast cancer are at increased risk of toxicity with HER2-targeted therapies and may require closer monitoring of toxicity. 

### 4.3. Newer HER2-Targeted Agents

Although newer HER2-targeted agents have been developed in the early stage and advanced setting for HER2-positive breast cancer, there are limited studies investigating the impact of BMI on the efficacy and toxicity of these agents. Pertuzumab is a monoclonal antibody that targets the HER2 receptor by binding to a different HER2 epitope inhibiting HER2 dimerization. Pertuzumab is administered at a fixed dose based on early clinical trials and population pharmacokinetic studies that show a clinically insignificant effect of body weight on pertuzumab distribution and clearance [80]. Similarly, the oral HER2-targeted tyrosine kinase inhibitors (TKIs)—lapatinib [81], neratinib [82], and tucatinib [83]—are also administered at fixed doses. On the other hand, the antibody-drug conjugates, ado-trastuzumab emtansine (T-DM1) and fam-trastuzumab deruxtecan-nxki (T-DXd), are dosed according to weight given that pharmacokinetic studies showed that body weight impacts both the volume of distribution and the clearance of these agents [84,85]. 

Although the safety and efficacy of the newer HER2-targeted agents have been investigated in early phase clinical trials, current evidence suggests that patients with obesity may have worse outcomes with these agents. In an observational study of 709 patients with metastatic HER2-positive breast cancer treated with pertuzumab and/or T-DM1, patients with obesity had worse OS (HR = 1.29, 95% CI 1.09–1.52, *p* = 0.003) compared to patients without obesity, but BMI had no impact on the PFS after first-line therapy (HR = 1.09, 95% CI 0.97–1.21, *p* = 0.15) [86]. In addition, in the NeoALTTO trial that randomized 455 patients to neoadjuvant lapatinib, trastuzumab, or their combination plus paclitaxel, patients who are overweight and those who are obese with HR-positive breast cancer were less likely to achieve a pathologic complete response (OR = 0.55, 95% CI 0.30–1.01, *p* = 0.053) compared to patients with normal weight [32]. However, this effect was not seen for HR-negative cases (OR 1.30, 95% CI 0.76–2.23, *p* = 0.331), which may reflect the poor prognostic impact of obesity in HR-positive breast cancer rather than a specific treatment-related effect.

Compared to trastuzumab, the newer HER2-targeted agents appear to be less cardiotoxic. For example, the addition of pertuzumab to trastuzumab plus docetaxel in the phase III CLEOPATRA trial did not increase the incidence of cardiac adverse events [87]. The antibody-drug conjugates, T-DM1 and T-DXd, and the oral HER2-targeted TKIs also have a relatively low incidence of cardiac toxicity compared to trastuzumab, although patients with a cardiac history or reduced LVEF at baseline were excluded from these trials. Nonetheless, patients with obesity may experience more adverse events with these newer HER2-targeted agents compared to patients without obesity. A retrospective study of 119 patients with HER2-positive breast cancer treated with T-DM1 found that patients with obesity treated with T-DM1 had a higher incidence of all-grade adverse events compared to patients without obesity, which resulted in a significantly higher rate of treatment modifications (45% vs. 25%, *p* = 0.028) and delays (36% vs. 16%, *p* = 0.015) [88]. To our knowledge, a similar analysis of toxicity according to BMI has not been performed for the more recent HER2-targeted agents. 

## 5. Targeted Therapies

### 5.1. CDK4/6 Inhibitors

Cyclin-dependent kinase (CDK) 4/6 inhibitors in combination with endocrine treatment are the standard-of-care treatment for first-line HR-positive metastatic breast cancer and are administered at fixed doses. CDK4 and CDK6 are serine/threonine kinases that regulate the cell cycle by forming cyclin D–CDK4/6 complexes, which leads to a downstream effect of cell cycle progression [89]. CDK4/6 inhibitors block the cell cycle transition from G1 to S by inhibiting the kinase activity of the cyclin D–CDK4/6 complex [89]. Preclinical data illustrate that CDK4 and CDK6 are also important regulators of metabolic processes, such as lipid synthesis, oxidative pathways, insulin signaling, glucose regulation, and mitochondrial function [90,91,92,93]. Likewise, CDK4/6 inhibitors also impact cellular metabolism, leading to changes in glycolysis and fatty acid oxidation, and subsequent apoptosis [94]. 

Based on the interaction of CDK4/6 inhibitors on cellular metabolism, the impact of obesity on the efficacy of CDK4/6 inhibitors is under investigation. The three CDK4/6 inhibitors, palbociclib, ribociclib, and abemaciclib, are each prescribed as a fixed dose based on population pharmacokinetic studies that show a clinically insignificant effect of body weight on drug exposure [95,96,97]. In the adjuvant setting, palbociclib showed no difference in invasive DFS outcomes for BMI groups (HR 0.95 CI 0.69–1.30) in the preplanned analysis of outcomes by BMI in the randomized, phase III PALLAS trial (comparing palbociclib with adjuvant endocrine therapy to adjuvant endocrine therapy alone in patients with early-stage breast cancer) [98]. In addition, patients who are overweight and those who are obese had less frequent and less severe neutropenia compared to patients with normal weight, which led to significantly lower treatment discontinuation rates with palbociclib (overweight vs. normal weight: HR = 0.73, 95% CI 0.63–0.84, *p* < 0.0001; obese vs. normal weight: HR = 0.65, 95% CI 0.56–0.75, *p* < 0.0001). In the neoadjuvant setting, an exploratory post hoc analysis of the NEOMONARCH trial, which compared the biologic activity of 2 weeks of neoadjuvant abemaciclib plus anastrozole, abemaciclib monotherapy, and anastrozole monotherapy followed by 14 weeks of the combination of abemaciclib and anastrozole in patients with early-stage breast cancer, showed that BMI (categorized by the threshold of 25) did not significantly impact Ki67% changes at 2 weeks or radiological and clinical response rates at the end of treatment with abemaciclib plus endocrine therapy [99]. 

In the metastatic setting, a pooled analysis from the MONARCH 2 and 3 trials, which compared abemaciclib plus endocrine therapy to endocrine therapy alone in patients with advanced breast cancer, found no difference in PFS according to BMI (*p* = 0.07). However, normal and/or underweight patients had a higher overall response rate with abemaciclib plus endocrine therapy compared with patients who are overweight and/or obese (49.4% vs. 41.6%, OR = 0.73, 95% CI 0.54–0.99) [100]. Similar to palbociclib in the PALLAS trial, patients who are overweight and those who are obese treated with abemaciclib in the MONARCH 2 and 3 trials had lower rates of neutropenia of any grade (40.4% vs. 51.0%, *p* = 0.004) and neutropenia grade 3 or higher (21.7% vs. 29.3%, *p* = 0.02) compared to underweight and/or normal weight patients [100]. The authors of this study propose that a potential mechanism of the decreased response rate to abemaciclib plus endocrine therapy observed in patients with obesity and metastatic disease may be due to a suboptimal dose intensity, but further prospective trials to evaluate this hypothesis are required. 

Retrospective studies evaluating the efficacy and toxicity of CDK4/6 inhibitors in metastatic disease are inconclusive. A study of 179 patients treated with palbociclib or ribociclib plus endocrine therapy reported that overweight patients tended to have a higher 12-month PFS compared to patients with normal weight and patients with obesity (72.2%, 52.9%, and 56.5%, respectively), although the results were not statistically significant (*p* = 0.054). In addition, toxicities of palbociclib and ribociclib plus endocrine therapy in this study were similar across BMI groups [101]. A retrospective cohort study of 222 patients treated with CDK4/6 inhibitors (208 received palbociclib, 7 received abemaciclib, and 7 received ribociclib) found no difference in PFS, toxicities, or treatment modifications according to BMI [102]. Another retrospective study of 50 patients treated with CDK4/6 inhibitors utilized computed tomography (CT)-based analyses of baseline body composition. While no significant differences in PFS were observed with higher BMIs, baseline sarcopenia was associated with worse PFS (20.8 vs. 9.6 months, HR = 2.52, 95% CI 1.02–6.19, *p* = 0.037), and patients with higher visceral fat indices and higher visceral fat density had better PFS (20.8 vs. 10.4 months, HR = 0.40, 95% CI 0.16–0.99, *p* = 0.041) [103]. This study suggests that certain body compositions may be an important indicator of CDK4/6 inhibitor efficacy.

### 5.2. mTOR/PI3K Inhibitors

Resistance to endocrine therapy in breast cancer is associated with the activation of the phosphatidylinositol 3-kinase (PI3K)–Akt-mammalian target of rapamycin (mTOR) intracellular signaling pathway. mTOR and PIK3 inhibitors inactivate mTOR and PIK3CA, respectively, to prevent the downstream signaling required for cell cycle progression and proliferation [104]. The PI3K–mTOR pathway also plays a central role in cellular metabolism. The PI3K–mTOR pathway is regulated by intracellular stimuli including nutrients and insulin. When mTOR is activated, downstream PI3K–Akt signaling is disrupted, which creates a negative feedback loop on insulin leading to insulin resistance [105,106]. Accordingly, mTOR inhibition restores insulin action on the PI3K–Akt pathway and prevents insulin-resistant effects of excess nutrients on insulin-mediated glucose transport in muscle and adipose cells [106]. Chronic treatment with an mTOR/PI3K inhibitor leads to dysregulated lipid and glucose metabolism, resulting in a host of metabolic side effects in patients [106,107]. 

The mTOR inhibitor everolimus is prescribed at a fixed dose based on pharmacokinetic studies that show no effect of body weight on pharmacokinetic characteristics in adults [108]. The randomized phase III BOLERO-2 trial showed the efficacy of everolimus in combination with exemestane in patients with ER-positive/HER2-negative advanced breast cancer resistant to AIs with a significant improvement in PFS (HR = 0.43, 95% CI 0.35–0.54, *p* < 0.001) compared to exemestane monotherapy [104]. A secondary analysis of the real-world, expanded-access, multi-center BALLET trial that investigated the safety of everolimus plus exemestane found no correlation between BMI at baseline and PFS (*p* = 0.38) [107]. Everolimus can cause significant weight loss with almost a quarter of patients losing more than 6.9% of their baseline weight, and patients with greater weight loss at the end of everolimus therapy had improved PFS compared to those without weight loss (grade 3 and 4 weight loss vs. grade 0: HR 0.69, 95% CI 0.48–0.99, *p* = 0.041). This suggests that everolimus-associated weight loss is a positive prognostic marker in patients with advanced HR-positive breast cancer and indicative of “on-target” toxicity. 

The PIK3CA inhibitor alpelisib is prescribed at a fixed dose based on pharmacokinetic studies that show no effect of body weight on pharmacokinetics [109]. The randomized, phase III SOLAR-1 trial of patients with PIK3CA-mutated HR-positive, HER2-negative advanced breast cancer who received prior endocrine therapy showed a longer PFS (HR = 0.65, 95% CI 0.50–0.85, *p* < 0.001) in patients treated with alpelisib plus fulvestrant compared with fulvestrant alone [110]. Similar to everolimus, weight loss is a common side effect of alpelisib, occurring in almost one-third of patients [110]. In addition, hyperglycemia frequently occurs with alpelisib treatment, with 65% of patients experiencing hyperglycemia of any grade and 36.6% of patients experiencing grade 3 or higher hyperglycemia [110]. Given that PI3K inhibitors block the intracellular action of insulin, hyperglycemia is considered another “on-target” effect [111].

There are limited data investigating the impact of obesity on the efficacy of alpelisib. One retrospective study of 27 patients with metastatic PIK3CA-mutated breast cancer treated with alpelisib showed no difference in response to alpelisib according to BMI (*p* = 0.966), although the study was limited by the small sample size [112]. Other studies have shown that patients with increased BMI, pre-existing hyperglycemia, and pre-diabetes/diabetes are at increased risk of alpelisib-induced hyperglycemia [113,114]. Management strategies have been proposed to prevent and treat alpelisib-induced hyperglycemia, including counseling on healthy lifestyle behaviors, optimization of blood glucose prior to alpelisib treatment, and treatment with anti-hyperglycemia agents [111]. Given that patients with obesity are at increased risk of alpelisib-induced hyperglycemia, these patients should be monitored carefully to allow for early detection and management of hyperglycemia and associated complications. 

### 5.3. PARP Inhibitors

The poly(ADP-ribose) polymerases (PARP) family of enzymes have a wide variety of biological functions, ranging from DNA repair, cell division and differentiation, oxidative stress, and cell death [115]. The PARP enzymes also regulate key metabolic pathways central to carbohydrate and lipid metabolism and adipocyte differentiation [115]. PARP inhibitors inactivate the PARP family of enzymes and are approved for use in the treatment of metastatic germline BRCA1 or BRCA2-mutated breast cancers and in the adjuvant setting for high-risk, HER2-negative germline BRCA1 or BRCA2-mutated early breast cancer [116,117,118]. The PARP inhibitors, olaparib and talazoparib, are both prescribed at fixed doses [119]. To our knowledge, no study has assessed the impact of BMI on the clinical outcomes and toxicities of PARP inhibitors in patients with breast cancer. 

### 5.4. Trop-2-Directed Antibody-Drug Conjugate

Sacituzumab govitecan-hziy is an antibody-drug conjugate that combines a humanized monoclonal antibody, which targets the human trophoblast cell-surface antigen 2 (Trop-2), with SN-38. Sacituzumab govitecan-hziy is approved for use in patients with relapsed or refractory metastatic triple-negative or HR-positive, HER2-negative breast cancer [120,121], and is dosed according to weight based on pharmacokinetic studies that show that body weight correlates with volume of drug distribution and clearance [122]. To our knowledge, no study has assessed the impact of BMI on the clinical outcomes and toxicities of sacituzumab govitecan-hziy in patients with breast cancer.

## 6. Immunotherapy

Pembrolizumab is a humanized monoclonal antibody that inhibits the PD-1 receptor and is approved for use in the treatment of patients with triple-negative breast cancer in the neoadjuvant and metastatic settings [123,124]. Although early clinical trials of pembrolizumab used a weight-based dosing regimen, subsequent pharmacokinetic studies show that a fixed-dosing strategy results in no clinically significant differences in efficacy and safety, supporting the current FDA recommendation of a fixed dose [125]. Data addressing the impact of obesity on clinical outcomes with immunotherapy are lacking. Although no study has investigated the impact of BMI with immunotherapy in breast cancer specifically, a systematic review of 18 studies that included many types of solid tumors (mainly non-small cell lung cancer, melanoma, and renal cell carcinoma) found mixed results with survival outcomes and immune-related adverse effects after treatment with immunotherapy [126]. Another systematic review of 13 studies treated with immune checkpoint inhibitors in solid tumors (also mainly non-small cell lung cancer, melanoma, and renal cell carcinoma) found a positive association between high BMI and improved OS (HR = 0.62, 95% CI 0.55–0.71, *p* < 0.0001) and PFS (HR = 0.71, 95% CI 0.61–0.83, *p* < 0.0001) among patients with immune checkpoint inhibitors, but no significant difference between the incidence of immune-related adverse effects (*p* = 0.207) [127]. 

It is hypothesized that patients with obesity and cancer may have a favorable effect with immunotherapy due to the immune dysfunction that results from excess adipose tissue. In breast cancer patients with obesity, the immune system is dysregulated with an increase in pro-inflammatory adipokines, such as CD8+, Th1 CD4+, and Th17 CD4+, and a decrease in anti-inflammatory adipokines, such as Th2 CD4+ and Tregs [128]. Obesity is also associated with an increase in myeloid-derived suppressor cells that fail to differentiate into mature myeloid lineages and impaired cytotoxic activity of T cells and NK cells. There is also increased macrophage recruitment in adipose tissue that results in the secretion of inflammatory cytokines and can further stimulate angiogenesis. In addition, obesity is associated with increased expression of PD-1 on T cells and NK cells and of PD-L1 on myeloid-derived suppressor cells. This suggests that breast cancer patients with obesity may experience a strong anti-tumor immune response from immunotherapy, which necessitates further confirmation through well-designed clinical trials.

## 7. ASCO Guidelines

ASCO published clinical practice guidelines in 2012 outlining recommendations for appropriate cytotoxic chemotherapy dosing for adult patients with cancer and obesity [18]. A systematic review of the literature was performed (with the majority of studies involving breast, ovarian, colon, and lung cancers) by a panel of experts in medical and gynecologic oncology, clinical pharmacology, pharmacokinetics and pharmacogenetics, and biostatistics and a patient representative to answer questions regarding chemotherapy dosing and toxicity in patients with obesity. More recently, ASCO published a guideline update in 2021 to provide recommendations on the appropriate dosing of immunotherapy and targeted cancer therapies in adults with cancer and obesity [19]. Sixty studies, primarily retrospective, were included in the review. 

According to ASCO guidelines, patients with obesity should be treated with full weight-based dosing of chemotherapy given that there is little evidence to suggest that toxicity is increased with full weight-based dosing and that underdosing is associated with inferior outcomes [19]. In addition, ASCO recommends that toxicities related to chemotherapy should be treated the same for all patients, regardless of obesity status. If a dose reduction is performed in response to toxicity, resumption of full weight-based doses for subsequent cycles should be considered, especially if a possible cause of toxicity (e.g., impaired renal or hepatic function) has resolved. For immunotherapy and targeted therapies, ASCO recommends the FDA-approved prescribing information for dosing, regardless of obesity status, given that there is little evidence to suggest that dosing strategies should be modified for patients with obesity. Dose reductions and modifications for immunotherapy and targeted therapies should be treated the same for all patients. 

## 8. Clinical Considerations for Patients with Obesity

Patients with breast cancer and obesity present multiple unique challenges during cancer treatment. Patients with obesity have different physiology and metabolism compared to patients without obesity, which leads to variable pharmacokinetics. In addition, patients with obesity are at higher risk for the development of diabetes mellitus and cardiovascular disease, which must often be weighed against the benefits of treatment. Furthermore, patients with obesity are more likely to have aggressive and advanced tumors and less likely to benefit from breast cancer treatment resulting in increased rates of relapse and death. Despite these important and unique considerations in managing patients with obesity, there are no specific guidelines for the management of breast cancer in patients with obesity. In the United States, the National Comprehensive Cancer Network (NCCN) guidelines are the most comprehensive guidelines detailing the standard-of-care for patients with breast cancer, but there is limited recommendations for patients with obesity specifically [129]. Therefore, we propose clinical considerations as outlined in Table 1 for the management of systemic therapies in breast cancer patients with obesity. 

## 9. Conclusions and Future Directions

Obesity is an independent poor prognostic factor for breast cancer patients, leading to increased breast cancer incidence, relapse, and mortality. Obesity can also impact the efficacy and toxicity of systemic therapies, which pose specific challenges during breast cancer treatment. Given the prevalence of obesity and the role that obesity plays on breast cancer risk and progression, further research investigating the impact of obesity on breast cancer is urgently needed (Table 2). A more detailed understanding of the metabolic pathways that drive breast cancer has the potential to identify biomarkers and lead to new targeted therapies. Lifestyle interventions and pharmacologic strategies for weight management are currently being investigated to reduce breast cancer risk and improve breast cancer outcomes. Clinical trials are ongoing to address the optimal therapy strategy in patients with obesity, but further trials are warranted. Given that patients with obesity are at increased risk of breast cancer recurrence and death, efforts to improve outcomes in patients with breast cancer and obesity should be a priority.

## Figures and Tables

**Table 1:** Systemic therapy concerns and considerations for patients with breast cancer and obesity.

Systemic Treatment	Mechanisms Related to Obesity	Dosing Strategy	Treatment Concerns in Patients with Obesity	Considerations for Patients with Obesity
Chemotherapy	BSA-based dosing strategies may not accurately estimate drug pharmacokinetics in patients with obesity [21] Chemotherapeutic agents can have different pharmacokinetic profiles in patients with obesity (e.g., lipophilic drugs with a high affinity for adipose tissue may have a higher volume of distribution in patients with obesity) [31,130]	BSA-based dosing	Risk of under- or over-dosing using BSA-based dosing formulas, which may lead to decreased efficacy and/or increased toxicity Leads to weight gain and cardiometabolic side effects	Use actual body weight in BSA-based dosing formulas
Endocrine therapy	Increased levels of estrogens due to aromatization of adipose tissue may lead to inadequate estrogen suppression with endocrine therapy [43,44] Dysfunctional adipocytes release adipokines, metabolites, and cytokines, which induce endocrine resistance by activating various signal transduction pathways, modulating apoptosis-related genes, and upregulating aromatase activity [14,45] Adipokines and cytokines have been found to directly diminish the efficacy of endocrine therapy in vitro [45] Increased insulin levels and IGF-1 activate the PI3K/AKT/mTOR and RAS/RAF/MAPK signaling pathway, leading to endocrine resistance [13,46,47] Chronic inflammation results in endocrine therapy resistance through the activation of proinflammatory molecules and reactive oxygen species [45,48,49,50]	Fixed-dose	Increased endocrine-related toxicities and joint symptoms in patients with obesity Leads to cardiometabolic side effects and increased risk of VTE in patients with obesity	Choice of endocrine therapy should be made irrespective of BMI Consider comorbidities and cardiac risk factors when evaluating endocrine therapy choice and duration in the adjuvant setting
Trastuzumab	Further research is needed	Weight-based (IV); fixed-dose (SC)	The first SC dose may be suboptimal Increased risk of cardiotoxicity and other adverse events in patients with obesity	Consideration of a loading dose with SC administration for patients who are overweight and those with obesity
Pertuzumab	Further research is needed	Fixed-dose	NA	Treat irrespective of BMI
Antibody-drug conjugates (T-DM1, fam-trastuzumab deruxtecan)	Further research is needed	Weight-based	Increased toxicity with T-DM1 in patients with obesity	Treat irrespective of BMI
Tyrosine kinase inhibitors (lapatinib, neratinib, tucatinib)	Further research is needed	Fixed-dose	NA	Treat irrespective of BMI
CDK4/6 inhibitors (palbociclib, ribociclib, abemaciclib)	CDK4 and CDK6 help regulate cellular metabolism, including lipid synthesis, oxidative pathways, insulin signaling, glucose regulation, and mitochondrial function [90,91,92,93]	Fixed-dose	NA	Treat irrespective of BMI
mTOR and PI3K inhibitor (everolimus and alpelisib, respectively)	Activation of the PIK3–mTOR pathway results in insulin resistance and altered glucose metabolism [105,106]	Fixed-dose	Associated with dyslipidemia, hyperglycemia (primarily alpelisib)	Obtain serial fasting blood sugars and lipid panels For alpelisib only: Optimize blood glucoses prior to initiation of alpelisib Counsel on healthy lifestyle behaviors and symptoms of hyperglycemia Closely monitor for signs and symptoms of hyperglycemia to allow for the early detection and management of hyperglycemia-related complications
PARP inhibitors (olaparib, talazoparib)	PARP enzymes help regulate metabolic pathways, including carbohydrate and lipid metabolism and adipocyte differentiation [115]	Fixed-dose	NA	Treat irrespective of BMI
Trop-2-directed antibody-drug conjugate (sacituzumab govitecan-hziy)	Further research is needed	Weight-based	NA	Treat irrespective of BMI
Immunotherapy (pembrolizumab)	Excess adipose tissue results in immune system dysfunction [128]	Fixed-dose	NA	Treat irrespective of BMI

BSA = body surface area; VTE = venous thromboembolism; IV= intravenous; SC = subcutaneous; NA = not applicable.

**Table 2:** Areas for future research in the prevention and treatment of obesity-associated breast cancer.

Prevention	Identifying the metabolic pathways that drive breast cancer for the development of biomarkers to predict those at increased risk Identifying the molecular mechanisms underlying the relationship between pre-menopausal patients and obesity that reduce breast cancer risk Improving risk prediction models that incorporate body composition parameters to predict breast cancer risk Improving lifestyle interventions and pharmacologic strategies for weight management for primary, secondary, and tertiary breast cancer prevention
Treatment	Identifying the metabolic pathways that drive breast cancer for the development of novel therapies and biomarkers that predict response to treatment Identifying targetable metabolic pathways that result in increased endocrine therapy resistance in patients with obesity Improving risk prediction models that incorporate body composition parameters to predict response to therapy Improving chemotherapy dosing strategies to reflect body composition in patients with obesity Optimizing the selection, dosing, and duration of hormone therapy for patients with obesity Improving strategies for the prevention and treatment of cardiometabolic adverse effects in patients with obesity Increasing the number of clinical trials focused on patients with obesity
